# Plasma chemokines CXCL10 and CXCL9 as potential diagnostic markers of drug-sensitive and drug-resistant tuberculosis

**DOI:** 10.1038/s41598-023-34530-z

**Published:** 2023-05-06

**Authors:** Pavithra Sampath, Anuradha Rajamanickam, Kannan Thiruvengadam, Alangudi Palaniappan Natarajan, Syed Hissar, Madhavan Dhanapal, Bharathiraja Thangavelu, Lavanya Jayabal, Paranchi Murugesan Ramesh, Uma Devi Ranganathan, Subash Babu, Ramalingam Bethunaickan

**Affiliations:** 1 Department of Immunology, ICMR-National Institute for Research in Tuberculosis (ICMR-NIRT), No.1. Mayor Sathyamoorthy Road, Chetpet, Chennai, 600 031 India; 2 ICMR-NIRT-NIH-International Center for Excellence in Research, Chennai, India; 3 Department of Statistics, ICMR-National Institute for Research in Tuberculosis (ICMR-NIRT), Chennai, India; 4 Department of Clinical Research, ICMR-National Institute for Research in Tuberculosis (ICMR-NIRT), Chennai, India; 5 Department of Clinical Pharmacology, ICMR-National Institute for Research in Tuberculosis (ICMR-NIRT), Chennai, India; 6 Greater Corporation of Chennai, Chennai, India; 7 Government Hospital for Thoracic Medicine (GHTM), Otteri, Chennai, India

**Keywords:** Immunology, Biomarkers, Diseases

## Abstract

Tuberculosis (TB) diagnosis still remains to be a challenge with the currently used immune based diagnostic methods particularly Interferon Gamma Release Assay due to the sensitivity issues and their inability in differentiating stages of TB infection. Immune markers are valuable sources for understanding disease biology and are easily accessible. Chemokines, the stimulant, and the shaper of host immune responses are the vital hub for disease mediated dysregulation and their varied levels in TB disease are considered as an important marker to define the disease status. Hence, we wanted to examine the levels of chemokines among the individuals with drug-resistant, drug-sensitive, and latent TB compared to healthy individuals. Our results demonstrated that the differential levels of chemokines between the study groups and revealed that CXCL10 and CXCL9 as potential markers of drug-resistant and drug-sensitive TB with better stage discriminating abilities.

## Introduction

Chemokines are the kick-starters of innate and adaptive immune responses by their chemotactic effects^1,2^. Numerous animal studies delineated the host protective and detrimental responses of chemokine ligands and their receptors during tuberculosis (TB). Chemokines govern containment of the causative agent *Mycobacterium tuberculosis* (MTB) through immune cell recruitment to the lung, granuloma formation^3^, DC migration and priming of T cells^4–6^. During TB, perpetuating inflammation leads to pathological shift and limits pathogen control, thus increasing the severity of the disease^7^. The devastating effects of TB are attributed to neutrophil accumulation with elevated levels of chemokine secretion^8,9^ and matrix metalloproteinases (MMP) that halt chemokine activity^10^ resulting in lung cavitation^7^. Genetic polymorphisms in C-C or C-X-C chemokine ligand genes also contribute to TB susceptibility^11–13^. Chemokines: CXCL8, CXCL9, CXCL10, CXCL11 and CXCL13 have been proposed to have a role in TB susceptibility and pathogenesis^7,14–17^.

Though TB is curable with a 6-month treatment regimen, it remains the second infectious disease of mortality next to COVID-19^18^. The slowly replicating MTB and their variants are successful invaders to gain survival resistance within the host effector milieu. TB management is crucial because of various reasons: (a) emergence of drug resistance either by patient non-compliance to drugs or due to the strain impact^19^; (b) difficulty in diagnosing LTB and TB progressors; (c) inability to discriminate different TB forms (LTB, drug-sensitive TB (DS-TB) and drug-resistant TB (DR-TB))^20^; (d) comorbidities; (e) longer treatment regimens^19^; (f) delayed culture diagnosis; (g) low sensitivity assays^21^ and so on. Host immune responses and soluble proteins are vital sources for understanding the pathobiology of infection to identify biomarkers. In the TB infection scenario, chemokines functionality and upregulation indicate their definitive roles as biomarkers for diagnosing active TB^22^ or latent TB (LTB)^23^, differentiating PTB from LTB^24^, bacterial burden and disease severity^25^, treatment monitoring^26^ and unfavourable outcomes^27^.

In continuum with this, we hypothesized that the behaviour of chemokines and their release in the host’s circulation during infection may vary between different forms of TB. In addition, their differential levels can reveal their biomarker efficiency in distinguishing TB from LTB or DR-TB. Therefore, we intended to estimate the circulating levels of C-C and C-X-C chemokine ligands by multiplex assay across the spectrum of TB infection (LTB, DS-TB, and DR-TB). Our results demonstrated the differences in the chemokine profile between the study groups. In addition, our results divulged the set of chemokines that could effectively discriminate HC, LTB, DS-TB, and DR-TB groups. Thus, our study aid in understanding the host immunological response in chemokine secretion towards different spectra of TB infection and deciphered the chemokine signature as potent biomarker targets.

## Methods

### Ethical approval and informed consent

The study was approved by the National Institute for Research in Tuberculosis Ethical committee (NIRT, IEC 2015022), Chennai, India. Informed consent was obtained from all the recruited individuals. All the experiments were performed in accordance with relevant guidelines and regulations.

### Study population

The study population consists of healthy controls (HC) (n = 40), latently infected individuals (LTB) (n = 40), drug-sensitive TB (DS-TB) (n = 40) and drug-resistant TB (DR-TB) (n = 40). The study cohort was same from our previous paper with detailed information of study population and sample characteristics^28^. Our cohort composed of adult participants aged above 18 and below 65 and are exclusive of other infections and co-morbid conditions. Clinically well characterized cohort that has been diagnosed for TB has been included in our study which are exclusive of other infections and other co-morbid conditions like Diabetes Mellitus, HIV, HCV and HBV. Blood was collected at one-time point from the DS-TB/DR-TB groups before starting treatment. Plasma was separated by centrifuging blood at 2600 rpm for 10 min and stored at − 80 °C until further assays.

### Multiplex assays

Circulating plasma levels of C-C and C-X-C chemokines were measured by Luminex Magpix Multiplex Assay system (Bio-Rad, Hercules, CA) using 10 plex Luminex Human Magnetic Assay kit (R&D systems) according to the manufacturer’s protocol. The tested 10 plex panel consists of the following chemokines: CCL1, CCL2, CCL3, CCL4, CCL11, CXCL1, CXCL2, CXCL9, CXCL10 and CXCL11.

### Statistical analysis

Graph-Pad PRISM Version 9.0 (GraphPad Software, CA, UA) was used to analyse the statistical difference among the groups. R software version 4.2.0 (R Core Team, 2022) was used to perform random forest analysis and principal component analysis. The Shapiro–Wilk normality test was performed to test the normality of the data. Chemokine concentrations are shown as median and interquartile ranges (IQR). Statistical significance between the study groups (DR-TB, DS-TB, LTB, and HC) for chemokine observations were analysed using the Dunn test corrected for multiple comparisons using Bonferroni’s test. Sensitivity and specificity were assessed using receiver operating curve (ROC) analysis. The importance of the chemokines was ranked through random forest (RF) analysis. The dimensional reduction was carried out using principal component analysis (PCA) to identify the classification pattern of the ranked chemokines. In order to measure the degree of association between the chemokines, Spearman coefficients were calculated. Hierarchical clustering was performed to visualize the segmentation of these chemokines in the study groups using the SOM module in the Multi-experiment Viewer Application (http://www.tm4.org/). *p* < 0.05 was considered statistically significant.

## Results

### Basic characteristics

The demographics and haematological data of the study participants with their significance values are described in detail in our previous paper^28^. The median age was 35 years (range 18–63) for DR-TB, 28 years (range 14–49) for DS-TB, 27 years (21–50) for LTB, and 27.5 years (range 18–50) for HCs were not significantly different between recruited individuals.

### Drug-resistant tuberculosis is associated with increased levels of chemokines

**Figure 1:**
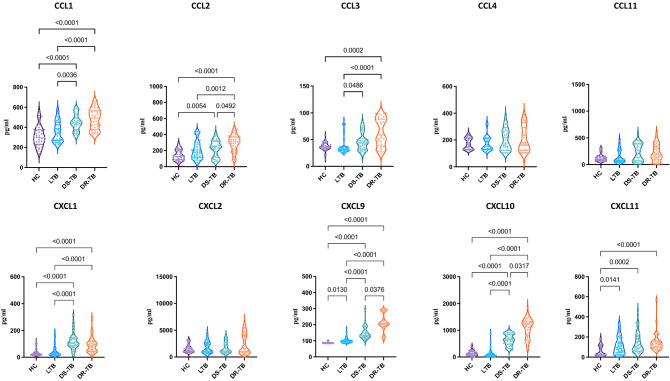
Altered chemokine profile among DS-TB and DR-TB groups compared to HC or LTB groups. Statistical differences were analysed by Dunn test corrected for multiple comparisons using Bonferroni test and significant *p* values < 0.05 were mentioned in the graphs.

We wanted to determine the dynamics of chemokines at the different spectra of TB disease and/or infection may therefore be useful as potential biomarker targets for diagnosis. We examined an array of CC and CXC chemokines using multiplex assay profiles in plasma of drug-resistant (DR-TB), drug-sensitive (DS-TB), and LTB and compared them with healthy controls. Chemokine concentration was shown as median and IQR in Table 1. As shown in Fig. 1, DR-TB exhibited significantly increased CC chemokines CCL2 (*p* = 0.0492), CXC chemokines CXCL9 (*p* = 0.376) and CXCL10 (*p* = 0.0317) in comparison to DS-TB.

**Table 1:** Chemokine concentration shown as median and interquartile range across the groups.

Chemokines	HC	LTB	DS-TB	DR-TB
CCL1	299.99 (236.11–371.56)	351.35 (272.5–433.42)	443.44 (378.14–491.03)	493.07 (393.03–558.91)
CCL3	36.08 (33.32–41.10)	32.59 (29.76–36.08)	43.93 (32.38–50.63)	58.16 (40.44–87.60)
CCL11	105.36 (77.68–153.94)	80.66 (249.73–190.18)	232.24 (78.75–386.97)	181.22 (107.34–327.48)
CXCL2	1298.54 (966.47–1803.76)	1371.12 (950.91–2668.26)	1253.85 (971.89–2549.04)	1521.06 (964.82–4182.35)
CXCL10	112.84 (62.75–198.59)	76.24 (42.56–148.35)	650.50 (483.88–840.13)	1205.75 (842.5–1322.25)
CCL2	129.72 (89.02–198.59)	176.17 (117.97–264.50)	244.4 (121.86–316.54)	312.48 (234.47–369.83)
CCL4	170.51 (129.61–211.65)	150.02 (129.61–211.75)	172.76 (122.77–266)	170.43 (122.77–292.93)
CXCL1	22.81 (18.22–36.03)	27.39 (16.96–53.06)	105.74 (84.88–142.44)	90.30 (45.64–115.13)
CXCL9	86.88 (84.22–88.66)	98.88 (90.43–104.88)	134.48 (125.79–160.33)	205.99 (189.11–224.39)
CXCL11	27.70 (21.21–115.51)	93.89 (45.98–162.21)	104.31 (61.59–194.7)	126.66 (98.19–159.59)

Further, DR-TB patients exhibited significantly increased levels of CC chemokines CCL1 (*p* < 0.0001), CCL2 (*p* = 0.0012), CCL3 (*p* < 0.0001), CXC chemokines CXCL1 (*p* < 0.0001), CXCL9 (*p* < 0.0001) and CXCL10 (*p* < 0.0001) in comparison to LTB individuals. DR-TB patients exhibited significantly higher levels of CCL1 (*p* < 0.0001), CCL2 (*p* < 0.0001), CCL3 (*p* = 0.0002), CXC chemokines CXCL1 (*p* < 0.0001), CXCL9 (*p* < 0.0001), CXCL10 (*p* < 0.0001) and CXCL11 (*p* < 0.0001) in comparison to the control group of individuals. DS-TB exhibited significantly higher levels of CCL1 (*p* = 0.0036), CCL3 (*p* = 0.0486), CXCL1 (*p* < 0.0001), CXCL9 (*p* < 0.0001) and CXCL10 (*p* < 0.0001) in comparison to individuals with LTB. DS-TB exhibited significantly higher levels of CCL1 (*p* < 0.0001), CCL2 (*p* = 0.0054), CXCL1 (*p* < 0.0001), CXCL9 (*p* < 0.0001), CXCL10 (*p* < 0.0001) and CXCL11 (*p* = 0.0002) compared to the control group of individuals. LTB individuals exhibited significantly increased levels of CXCL9 (*p* = 0.0130) and CXCL11 (*p* = 0.0141) in comparison to the control group of individuals. Thus, the clinical spectrum of TB disease/ infection is associated with increased levels of chemokines.

### Heatmaps divulge tendencies in the chemokine milieu in DR-TB, DS-TB, LTB, and HC

**Figure 2:**
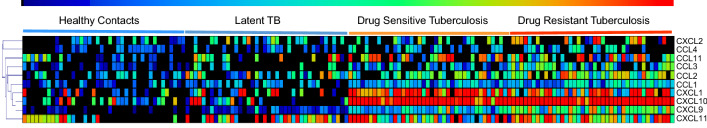
Heatmaps representing the measured chemokines and their hierarchical clustering across the TB disease spectrum by log 2 conversion and HC group mean normalization.

The trends in the chemokine expression profile were assessed by hierarchical clustering of chemokines using normalized values. For this, the raw individual chemokine expression counts were transformed to log 2 values and normalized with group mean value of HC for respective chemokine across all the samples. The normalized counts were shown in Fig. 2, and the color panel of the heat map reveals the serial increase of chemokines (both numbers and levels) from latency (black or blue) to drug-sensitive (blue, green, and red) and to drug-resistant TB (blue, green, orange, and red). Before infection, the latent condition presented the chemokine panel with near-high levels of CXCL11 and mild or moderate levels of CXCL2, CCL4, CCL2, CCL1, and CXCL9. In the diseased state, DS-TB individuals presented differential chemokine expression with high levels of CXCL1 and CXCL10; near high levels of CCL11, CCL2 and CXCL11; moderate levels of CCL1 and CXCL9 and mild levels of CXCL1, CCL4 and CCL3. DR-TB individuals are associated with abundant chemokine expression with high levels of CXCL1 and CXCL10; near high levels of CXCL2, CCL11, CCL3, CCL2, CCL1, CXCL9 and CXCL11 and with moderate expression of CCL4. Thus, these analyses help to reveal the power of chemokines to demarcate the spectrum of TB disease/infection (DR-TB, DS-TB, and LTB) from HC.

### Diagnostic performance of the top chemokines for the bifurcation of DR-TB, DS-TB, LTB and HC

**Figure 3:**
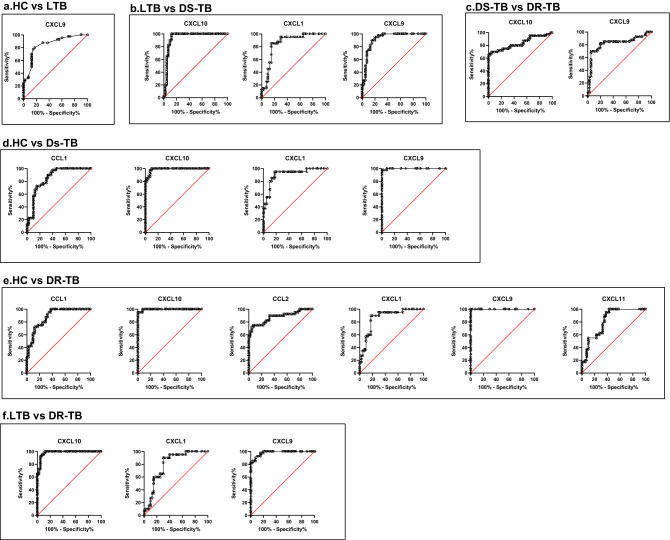
ROC curves of significant chemokines with AUC > 0.8 showing the diagnostic efficiency between the study groups, (**a**) HC vs LTB, (**b**) LTB vs DS-TB, (**c**) DS-TB vs DR-TB, (**d**) HC vs DS-TB, (**e**) HC vs DR-TB and (**f**) LTB vs DR-TB.

We conducted a ROC analysis of single variables to determine the diagnostic capabilities of each chemokine to distinguish between the study groups. Representative curves showing the chemokines with the best diagnostic precision between and among these groups are shown in Fig. 3, CXCL9 (AUC = 0.82, *p* < 0.0001) and CXCL10 (AUC = 0.84, *p* < 0.0001) discriminate DR-TB from DS-TB. Chemokines such as CXCL1 (AUC = 0.80, *p* < 0.0001), CXCL9 (AUC = 0.98, *p* < 0.0001) and CXCL10 (AUC = 0.98, *p* < 0.0001) discriminate DR-TB from LTB. Additionally, the chemokines CCL1 (AUC = 0.88, *p* < 0.0001), CCL2 (AUC = 0.88, *p* < 0.0001), CXCL1 (AUC = 0.87, *p* < 0.0001), CXCL9 (AUC = 1, *p* < 0.0001), CXCL10 (AUC = 1, *p* < 0.0001) and CXCL11 (AUC = 0.82, *p* < 0.0001) discriminate DR-TB from HC group. CXCL1 (AUC = 0.85, *p* < 0.0001), CXCL9 (AUC = 0.92, *p* < 0.0001) and CXCL10 (AUC = 0.94, *p* < 0.0001) discriminate DS-TB from individuals with LTB. CCL1 (AUC = 0.85, *p* < 0.0001), CXCL1 (AUC = 0.91, *p* < 0.0001), CXCL9 (AUC = 1, *p* < 0.0001) and CXCL10 (AUC = 0.99, *p* < 0.0001) discriminate DS-TB from the HC group of individuals. CXCL9 (AUC = 0.8503, *p* < 0.0001) discriminate the LTB individuals from the HC group of individuals. However, other chemokines CCL3, CCL4, CCL11 and CXCL2 could not significantly discriminate DR-TB from DS-TB, LTB, and the control group.

**Figure 4:**
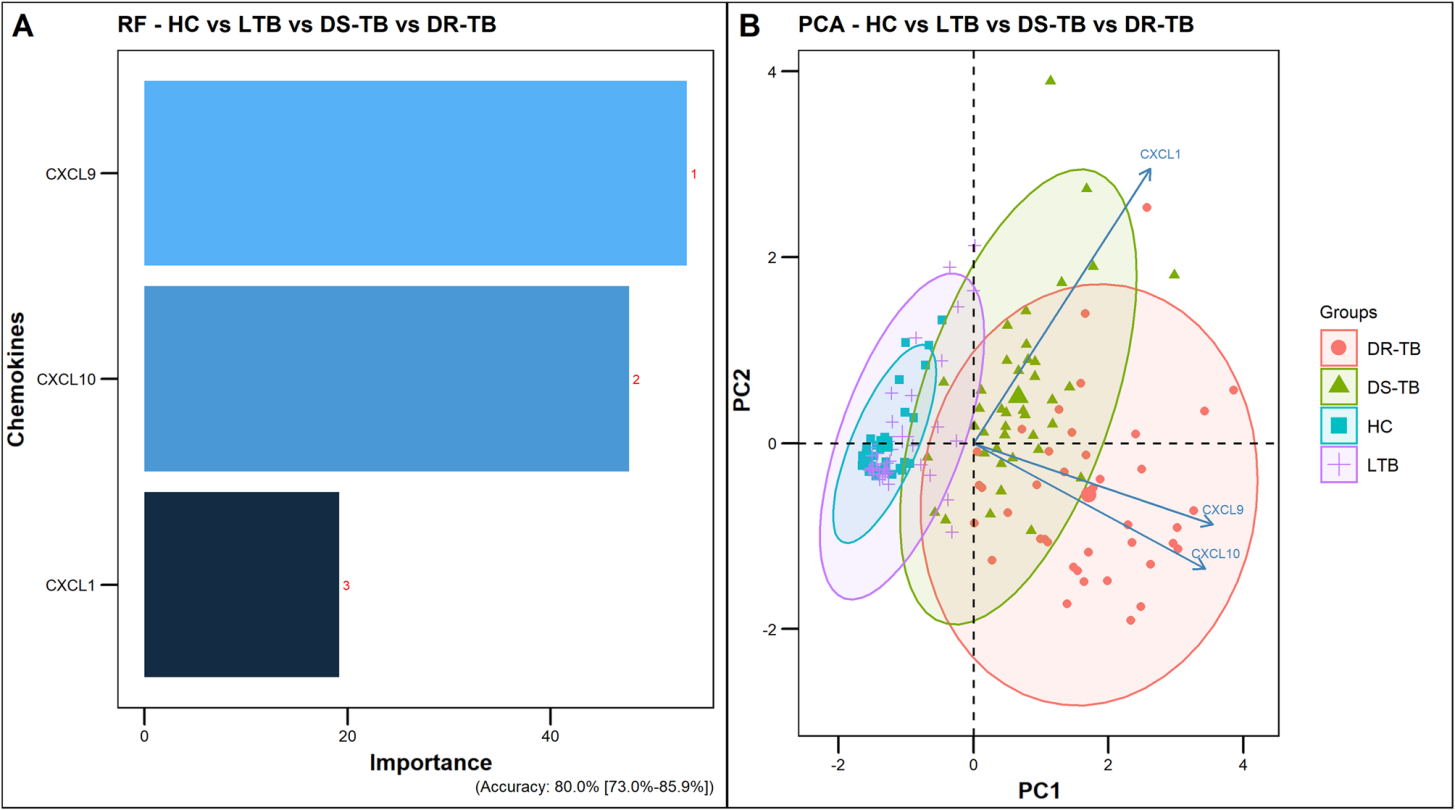
Random-forest analysis plot (**A**) and principal component analysis plot (**B**) of top 3 chemokines across the study groups (HC vs LTB vs DS-TB vs DR-TB).

**Figure 5:**
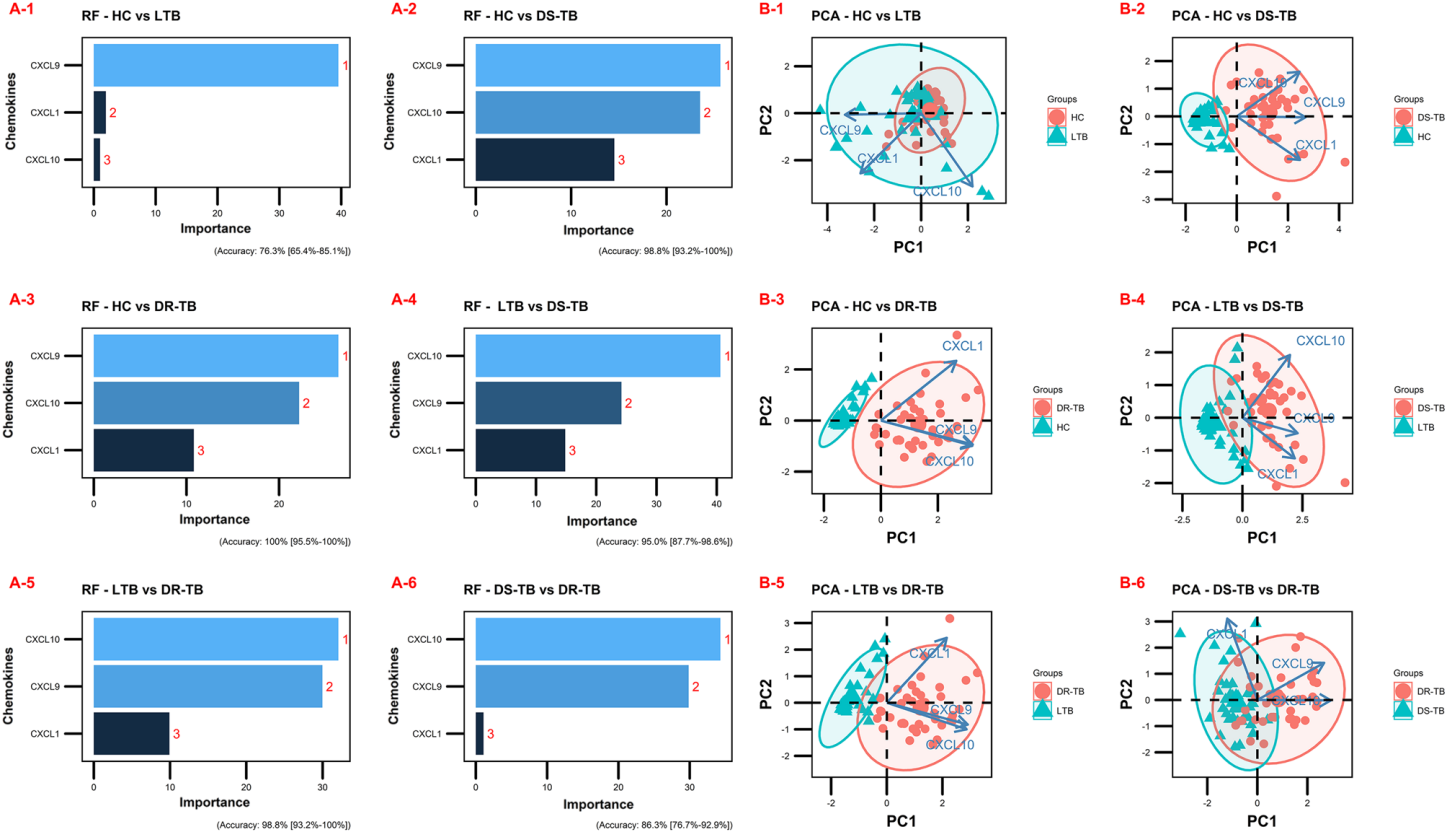
Sub-group comparisons of top 3 chemokines by random-forest analysis (**A1** HC vs LTB, **A2** HC vs DS-TB, **A3** HC vs DR-TB, **A4** LTB vs DS-TB, **A5** LTB vs DR-TB and **A6** DS-TB vs DR-TB) and principal component analysis (**B1** HC vs LTB, **B2** HC vs DS-TB, **B3** HC vs DR-TB, **B4** LTB vs DS-TB, **B5** LTB vs DR-TB and **B6** DS-TB vs DR-TB).

**Figure 6:**
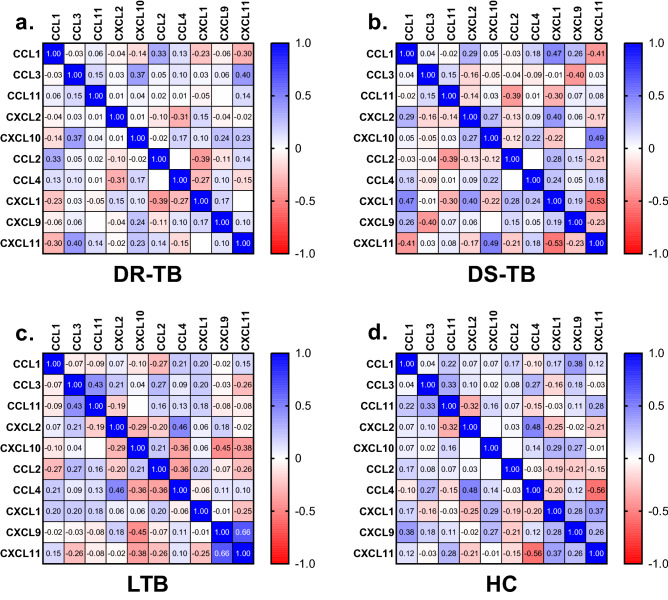
Correlation matrix using spearman rank correlation between the measured chemokines of the study groups, (**a**) DR-TB, (**b**) DS-TB, (**c**) LTB and (**d**) HC.

In addition, we performed a random forest (RF) analysis to understand the importance of these chemokines and their distinguishing ability toward the separation of study groups. According to the order of importance, RF plots of overall comparison (HC vs LTB vs DS-TB vs DR-TB) presented CXCL9, CXCL10, and CXCL1 as the top classifiers (Fig. 4A). This was in accordance with the ROC results where these chemokines displayed higher AUC values of above 0.8. Similarly in the subgroup comparisons, the same CXCL9 was obtained as the top classifier for HC vs LTB/DS-TB/DR-TB (Fig. 5A-1–A-3) whereas, CXCL10 for LTB vs DS-TB/DR-TB (Fig. 5A-4,A-5) and DS-TB vs DR-TB (Fig. 5A-6).

All chemokine variables were then dimensionally reduced through the principal component analysis, resulting in a lower variation of the first two dimensions, and the ellipses of HC overlapped within LTB and DS-TB within DR-TB. To achieve better bifurcation with a minimum of 80% variance, the weaker chemokines from the RF plots were removed and the dimensionality reduction analysis was repeated. The PCA of the top 3 chemokines (CXCL9, CXCL10 and CXCL1) exhibited better separation of clusters (LTB, DS-TB, and DR-TB) with variances above 80% (Fig. 4B). However, HC overlapped completely within the LTB cluster. The discriminative accuracy and the ranges are as follows: 80% (73–85.9%) for HC vs LTB vs DS-TB vs DR-TB and for subgroup comparison with a descending accuracy order of 100% (95.5–100%) for HC vs DR-TB; 98.8% (93.2–100%) for LTB vs DR-TB and HC vs DS-TB; 95% (87.7–98.6%) for LTB vs DS-TB; 86.3% (76.7–92.9%) for DS-TB vs DR-TB and the least 76.3% (65.4–85.1%) for HC vs LTB (Fig. 5B-1–B-6).

### Correlation between plasma chemokines and spectrum of TB infection/disease

For each pair of chemokines, Spearman’s rank correlation coefficient was calculated to determine the strength of association. There was a positive moderate correlation between the following pairs of chemokines in DR-TB: CXCL10 and CCL3 (r = 0.4, *p* = 0.0176), CXCL11 and CCL3 (r = 0.4, *p* = 0.0111), CCL2 and CCL1 (r = 0.3, *p* = 0.0388); DS-TB: CXCL1 and CCL1 (r = 0.5, *p* = 0.0024), CXCL10 and CXCL11 (r = 0.5, *p* = 0.0014), CXCL1 and CXCL2 (r = 0.4, *p* = 0.0112); LTB: CCL4 and CXCL2 (r = 0.5, *p* = 0.0027), CCL11 and CCL3 (r = 0.4, *p* = 0.0050); and HC: CCL4 and CXCL2 (r = 0.5, *p* = 0.0018), CXCL9 and CCL1 (r = 0.4, *p* = 0.0143), CXCL11 and CXCL1 (r = 0.4, *p* = 0.0195), CCL11 and CCL3 (r = 0.3, *p* = 0.0381). Whereas, there was a negative moderate correlation between the following pairs of chemokines in DR-TB: CXCL1 and CCL2 (r = − 0.4, *p* = 0.0136), CCL4 and CXCL2 (r = − 0.3, *p* = 0.0478); DS-TB: CXCL11 and CXCL1 (r = − 0.5, *p* = 0.0004), CXCL11 and CCL1 (r = − 0.4, *p* = 0.0092), CXCL9 and CCL3 (r = − 0.4, *p* = 0.0095), CCL11 and CCL2 (r = − 0.4, *p* = 0.0119); LTB: CXCL9 and CXCL10 (r = − 0.4, *p* = 0.0039), CXCL11 and CXCL10 (r = − 0.4, *p* = 0.0159), CCL4 and CXCL10 (r = − 0.4, *p* = 0.022), CCL4 and CCL2 (r = − 0.4, *p* = 0.0205); and HC: CXCL11 and CCL4 (r = − 0.6, *p* = 0.0001), CXCL2 and CCL11 (r = − 0.3, *p* = 0.0448) shown in Fig. 6a–d.

## Discussion

Understanding the host immune response upon infection is a key for unlocking disease pathogenesis and recognizing the protective or pathological linkers. During infection, the coaction of inflammatory signals and chemokines determines their levels and timing of production, thus mediating protection or causing damage to the host^7,25^. MTB infection rooted higher levels of chemokines in the circulation of PTB patients^25^ and their circulating levels are described as discriminators for LTB and active TB^29^. However, the nature of individuals infected with DR-TB is less focused. Our hypothesis is that individuals with DR-TB may have different chemokine profiles compared to those with DS-TB. To understand this, we considered having an overview of C-C and C-X-C chemokines across the TB spectrum (LTB, DS-TB, and DR-TB) and healthy individuals, thereby identifying biomarker targets, especially between DR-TB vs DS-TB, DS-TB vs LTB and LTB vs HC comparisons. Our results demonstrated two main findings: (i) differential levels between groups (moderate increase in LTB, high in DS-TB, and extremely high in DR-TB) compared to HC and (ii) chemokines (CXCL10, CXCL9 and CXCL1) as the promising biomarker signature. These findings correlate with the previous postulations as both CXCL10 and CXCL9 and their elevated levels has been considered as potent diagnostic markers for both pulmonary and extrapulmonary TB^2,25,30,31^.

Decades of research have been invested in CXCL10 to outperform the sensitivity issues faced through IGRA. CXCL10 are induced by antigen-presenting cells (APC) and stimulated macrophages during infection that assist chemotaxis, leukocyte migration, cell growth and angiogenesis and recent data determined that it could also restrict MTB replication^32–37^. CXCL10/IP-10 alone or in combination with acute phase proteins or cytokines are proposed as markers of bacterial burden^25^, culture conversion^25,38^, LTB and active TB discrimination^23,37^, treatment response^27,34,38,39^, childhood TB diagnosis^40^ and triage test for TB diagnosis^41^. Ferrian et al., reported lower CXCL10 levels in DR-TB that contrasted the previous reports from DS-TB and the authors claimed it as immunological suppression due to continuous TB exposure and re-treatment^38^. However, in our study DR-TB samples are of a similar kind, which are previously treated for TB but had strikingly higher CXCL10 than DS-TB, LTB, and HC individuals. This could possibly be due to the infiltrating APCs and associated hyper-inflammation that aids disease severity. Furthermore, the disease-mediated elevation of CXCL10 is evident, as it stands out as the top classifier for DR-TB vs DS-TB/LTB and DS-TB vs LTB.

CXCL9 came out as the topmost for discriminating DR-TB/DS-TB from HC. CXCL9 is an IFN-gamma-induced chemokine that is predominantly elevated in diseased groups in our study. These are crucial drivers for T cell recruitment and activation and their reduced levels in BAL fluids of infants’ alveolar macrophages are suggestive of less protection against H37Rv infection^42^. Along with IFN-gamma, CXCL9 can determine the disease severity upon ESAT-6 induction^43^ and their increased concentration declines after therapy^44^. The other promising candidates reported were CCL1, CCL3, and CXCL1^25^. Among which, CXCL1 emerged as a potential candidate that effectively discriminates active TB from LTB, healthy and non-TB lung disease and successfully met the WHO’s target product profile (TPP) criteria^45^. Although CXCL1 emerges one among the top 3 chemokines in our study, it only had the statistical significance to distinguish the diseased state (DS-TB/DR-TB) from healthy or LTB and did not discriminate DR-TB from DS-TB or LTB from HC.

Our data revealed that most of the estimated chemokines CXCL11, CXCL10, CXCL9, CXCL1, CCL3, CCL2, and CCL1 were remarkably higher in the DR-TB and DS-TB groups. Thus, many chemokines invariably extend their association towards disease burden with good diagnostic abilities with profound AUC values greater than 0.8 between various group comparisons. Nevertheless, only a few were able to differentiate DR-TB from DS-TB. Interestingly, Guzman et al., stated the probable differences between MDR-TB and DS-TB are due to the expression pattern of chemokine receptors (CCR2 and CCR4) in monocytes that control the kinetics of immune cell migration and recruitment rather than chemokine ligands. They also observed a continued increase of CD3+ monocytes, CCR4+ monocytes, CXCR1+ and CXCR3+ T cells in the circulation of MDR-TB patients even after anti-TB treatment that could aid chronicity of infection and delay in recovery^46^. Being a cross-sectional approach, our study lacked the follow-up data that could promptly help to identify biomarkers for culture conversion, bacterial burden, treatment response and outcome. In addition to this, the investigated panel had limited chemokines and other prominent chemokines (for example CCL-5) are being missed out. However, on a positive side, our study had an appreciable sample size with the inclusion of different spectra of TB infection/disease (LTB, DS-TB, and DR-TB groups) that could briefly suggest the chemokine signature with their diagnostic abilities. To conclude, CXCL10 and CXCL9 emerged as signatures for drug-sensitive and drug-resistant TB. Further extending the chemokine panel with longitudinal and functional studies may enable the true candidates to diagnose different TB infection stages.

## Data Availability

The data supporting the findings of this article will be made available by the corresponding author, upon request.
